# Deep learning in fracture detection: a narrative review

**DOI:** 10.1080/17453674.2020.1723292

**Published:** 2020-02-05

**Authors:** Pishtiwan H S Kalmet, Sebastian Sanduleanu, Sergey Primakov, Guangyao Wu, Arthur Jochems, Turkey Refaee, Abdalla Ibrahim, Luca v. Hulst, Philippe Lambin, Martijn Poeze

**Affiliations:** a Maastricht University Medical Center+, Department of Trauma Surgery, Maastricht;;; b The D-Lab: Decision Support for Precision Medicine, GROW—School for Oncology and Developmental Biology, Maastricht University Medical Center+, Maastricht;;; c Department of Radiology and Nuclear Medicine, GROW – School for Oncology and Developmental Biology, Maastricht University Medical Centre+, Maastricht, The Netherlands;;; d Division of Nuclear Medicine and Oncological Imaging, Department of Medical Physics, Hospital Center Universitaire De Liege, Liege, Belgium;;; e Department of Nuclear Medicine and Comprehensive diagnostic center Aachen (CDCA), University Hospital RWTH Aachen University, Aachen, Germany.;; f Nutrim School for Nutrition, Toxicology and Metabolism, Maastricht University, Maastricht, The Netherlands

Correction of conflict of interest statement

*Original statement:* The authors declare that they have no conflict of interest.

Corrected version:

Conflict of interest

The authors, except dr. Philippe Lambin, declare that they have no conflict of interest.Dr. Lambin reports, within and outside the submitted work, grants/sponsored research agreements from Varian medical, Oncoradiomics, ptTheragnostic, Health Innovation Ventures and DualTpharma. He received an advisor/presenter fee and/or reimbursement of travel costs/external grant writing fee and/or in kind manpower contribution from Oncoradiomics, BHV, Merck and Convert pharmaceuticals. Dr Lambin has shares in the company Oncoradiomics SA and Convert pharmaceuticals SA and is co-inventor of two issued patents with royalties on radiomics (PCT/NL2014/050248, PCT/NL2014/050728) licensed to Oncoradiomics and one issue patent on mtDNA (PCT/EP2014/059089) licensed to ptTheragnostic/DNAmito, three non-patentable invention (softwares) licensed to ptTheragnostic/DNAmito, Oncoradi­omics and Health Innovation Ventures.

